# Structure and Functional Analysis of the RNA- and Viral Phosphoprotein-Binding Domain of Respiratory Syncytial Virus M2-1 Protein

**DOI:** 10.1371/journal.ppat.1002734

**Published:** 2012-05-31

**Authors:** Marie-Lise Blondot, Virginie Dubosclard, Jenna Fix, Safa Lassoued, Magali Aumont-Nicaise, François Bontems, Jean-François Eléouët, Christina Sizun

**Affiliations:** 1 Unité de Virologie et Immunologie Moléculaires (UR892), INRA, Jouy-en-Josas, France; 2 Institut de Chimie des Substances Naturelles, CNRS UPR 2301, Gif-sur-Yvette, France; 3 IBBMC, Université Paris-XI, Orsay, France; Harvard Medical School, United States of America

## Abstract

Respiratory syncytial virus (RSV) protein M2-1 functions as an essential transcriptional cofactor of the viral RNA-dependent RNA polymerase (RdRp) complex by increasing polymerase processivity. M2-1 is a modular RNA binding protein that also interacts with the viral phosphoprotein P, another component of the RdRp complex. These binding properties are related to the core region of M2-1 encompassing residues S58 to K177. Here we report the NMR structure of the RSV M2-1_58–177_ core domain, which is structurally homologous to the C-terminal domain of Ebola virus VP30, a transcription co-factor sharing functional similarity with M2-1. The partial overlap of RNA and P interaction surfaces on M2-1_58–177_, as determined by NMR, rationalizes the previously observed competitive behavior of RNA versus P. Using site-directed mutagenesis, we identified eight residues located on these surfaces that are critical for an efficient transcription activity of the RdRp complex. Single mutations of these residues disrupted specifically either P or RNA binding to M2-1 *in vitro*. M2-1 recruitment to cytoplasmic inclusion bodies, which are regarded as sites of viral RNA synthesis, was impaired by mutations affecting only binding to P, but not to RNA, suggesting that M2-1 is associated to the holonucleocapsid by interacting with P. These results reveal that RNA and P binding to M2-1 can be uncoupled and that both are critical for the transcriptional antitermination function of M2-1.

## Author Summary

Premature termination of transcription by the RNA-dependent RNA polymerase (RdRp) complex of respiratory syncytial virus (RSV) is prevented by the M2-1 protein. This transcription factor interacts with both RNA and viral phosphoprotein P, the main RdRp cofactor, through a specific “core” domain. Using NMR, we solved the 3D structure of this domain and characterized the surface residues involved in P- and RNA-binding. Based on these data, we designed point mutations impairing binding to either RNA or P. We studied the functional implications of these mutations for transcription and co-localization of full-length M2-1 in cytoplasmic inclusion bodies, where viral transcription likely occurs. We found that the RNA- and P-binding surfaces are in close proximity, accounting for competitive binding *in vitro*. Our results suggest that binding to both RNA and to P is necessary for transcriptional antitermination by M2-1, but that the role of the interaction with P is primarily to recruit M2-1 to the RdRp complex. Finally we show that the M2-1 core domain is homologous to the C-terminal domain of Ebola virus VP30 despite low sequence identity, solidifying the relationship between these two proteins and transcriptional regulation strategies shared by viruses belonging to the *Filoviridae* family and the *Pneumovirinae* subfamily.

## Introduction

Human respiratory syncytial virus (RSV), a pneumovirus of the *Paramyxoviridae* family in the *Mononegavirales* order, is an important respiratory pathogen and the major cause of bronchiolitis and pneumonia in children [1]. Bovine RSV on the other hand represents an important economic issue due to the high morbidity and mortality of infected calves [2]. Whereas current efforts are mainly focused on the development of safe RSV vaccines for infants, the development of antiviral drugs specifically targeting viral-specific functions such as the RSV RNA-dependent RNA polymerase complex (RdRp) represents a promising alternative for treatment.

Four of the 11 proteins (the nucleoprotein N, the phosphoprotein P, M2-1 and the large polymerase subunit L), encoded by the RSV single-stranded negative-sense genomic RNA, are associated with the viral genome to form the holonucleocapsid [3]. The genomic RNA of RSV is maintained as a nuclease-resistant N-RNA ribonucleoprotein complex, which acts as a template for the RdRp that is responsible for both replication and transcription of the genome. Whereas the highly processive replicase generates a complete positive-sense RNA, which acts in turn as a template for genomic RNA synthesis, the transcriptase produces ten different subgenomic capped and polyadenylated mRNAs. Transcription proceeds by a sequential stop-and re-start mechanism in which the polymerase responds to *cis*-acting signals present in intergenic regions [4]. Transcription is (re)initiated at a highly conserved 9–10 nucleotide transcription promoter (gene start, GS) signal. Semi-conserved 12–13 nucleotide gene ends (GE) signal for polyadenylation and release of the nascent mRNA [5]. The polymerase has a propensity to dissociate from the N-RNA template, but cannot reinitiate at a downstream gene in case of premature termination [6], which leads to a decreasing transcription gradient from the 3′ to the 5′ end of the genome.

For all known pneumoviruses, RdRp driven transcription depends on M2-1. RSV M2-1 is a transcription antitermination factor that is important for the efficient synthesis of full-length mRNAs [7] as well as for the synthesis of polycistronic readthrough mRNAs [4], [6], [8], [9]. The latter activity is thought to facilitate polymerase access to promoter-distal regions of the genome, and hence transcription of all genes [3]. It was shown that the M2-1 protein reduced termination at all gene junctions, but that the efficiency in the presence of M2-1 varied at the different gene junctions [6], [9]. However, mechanisms by which M2-1 prevents the polymerase from terminating transcription remain to be clarified. There are at least three different scenarios. (i) M2-1 could bind to the nascent mRNA transcript to facilitate transcription elongation, perhaps by preventing the mRNA from re-hybridizing to the template, or forming secondary structures that might destabilize the transcription complex. This hypothesis is sustained by the finding that RSV mRNA are co-precipitated with M2-1 from RSV infected cells [10]. (ii) The polymerase processivity enhancing effect of M2-1 could be due to an increase of the affinity of the polymerase for the genomic RNA template in a sequence non-specific manner. (iii) M2-1 could recognize GE sequences either on the nascent mRNA or on the RNA template and prevent the release of the polymerase complex from its template, favoring transcription re-initiation at the downstream GS sequences.

In RSV-infected cells, M2-1 co-localizes with the other RdRp components in inclusion bodies (IBs) [11], which are regarded as centers of RNA synthesis [12]. The basic 194-residue RSV M2-1 has been shown to be an RNA binding protein, but specificity of M2-1 RNA binding has been debated. It was reported that M2-1 was able to bind to long RNAs without sequence specificity and with an apparent K_d_ of 30 nM, and that it bound specifically to short (80 nucleotides) but not long (700 nucleotides) RNAs containing the positive-sense antigenomic leader sequence with an apparent K_d_ of 90 nM [13]. Elsewhere it was demonstrated that M2-1 interacts more specifically with viral mRNAs not containing the leader sequence during infection [10]. In addition M2-1 interacts with P *in vitro* [14], competitively to RNA [15]. M2-1 is a modular protein that contains four domains. M2-1 forms tetramers in solution [15], [16], and the oligomerization domain was mapped to the region 33–62 [15]. The N-terminal region (residues 1–30) contains a putative zinc binding domain with a Cys_3_His motif, which is essential for the function of M2-1, but whose exact role is still unknown [17], [18], [19]. The RNA and P binding properties are related to the central part (or core domain) of the molecule (residues 53–177) [13], [15]. The C-terminal tail is predicted to be unstructured.

Here we have addressed the molecular basis of the interaction between RSV M2-1 and its partners. We have investigated the structural aspects of P and RNA binding to the core domain of M2-1 by NMR spectroscopy and report the solution structure of RSV M2-1_58–177_, which shows structural homology with the C-terminal domain (CTD) of the Ebola virus (EBOV) VP30 protein. In this α-helical domain we have identified residues that contribute to two adjacent, partially overlapping contact surfaces, with P and RNA respectively. We show that mutations of several of these residues specifically disrupt the M2-1:P or M2-1:RNA interaction *in vitro* and have a drastic effect on intracellular co-localization of full-length M2-1 with P as well as on the function of M2-1 as a transcription co-factor.

## Results

### Solution NMR structure of RSV M2-1_58–177_

**Figure 1:**
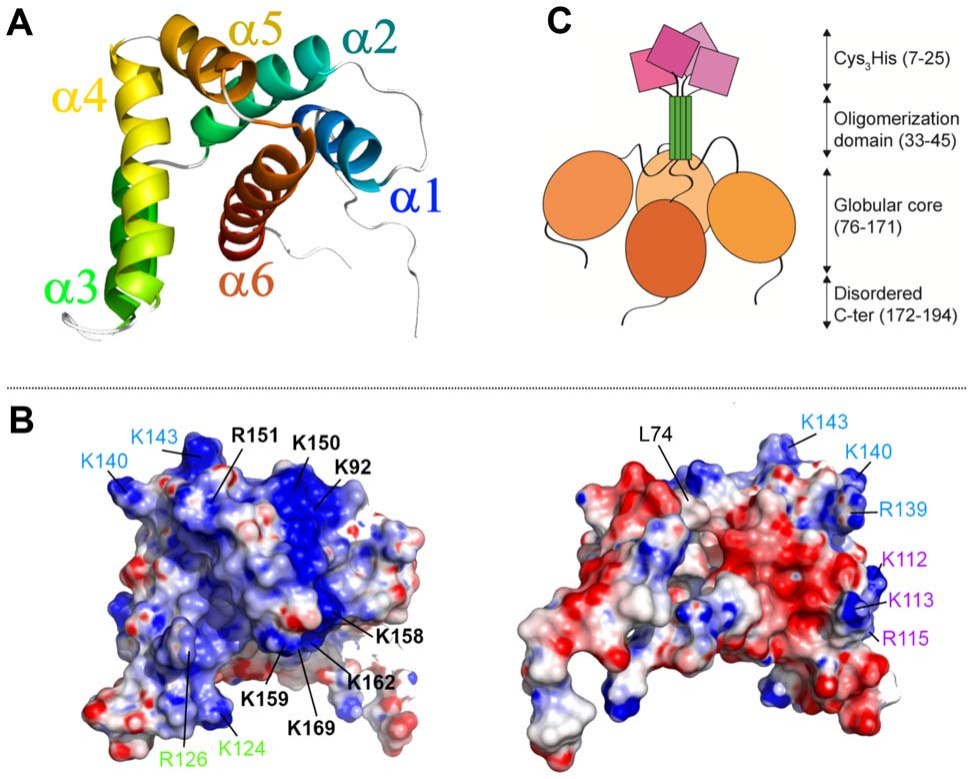
Solution structure of the core domain of RSV M2-1. (A) Cartoon representation of the NMR structure of the α-helical domain of M2-1_58–177_ (model of lowest energy). The color is ramped from blue to red from the N- to the C-terminus. (B) Electrostatic surface potential of M2-1_58–177_ calculated with DELPHI [58] using PARSE parameters [59]. Two opposite faces of the protein are shown. The left-hand view is the same as for panel A. Colors for charges are red to blue for potential energies −6 to +6 k_B_T. Basic residues belonging to the main cluster are labeled in black bold letters. Basic residues belonging to the 3 minor clusters are indicated in blue, green and purple letters. (C) Schematic representation of the tetramer of full-length M2-1.

The boundaries of the protein fragment M2-1_58–177_ were chosen to focus on the binding regions of RNA and RSV phosphoprotein determined previously, but also to exclude the oligomerization domain and the disordered C-terminus, which are not necessary for the interactions with RNA and P [15]. Line widths of the solution NMR spectra were compatible with a monomeric state, and M2-1_58–177_ was amenable to structure determination by NMR, in contrast to tetrameric full-length M2-1. The resonance assignments were reported elsewhere [20]. M2-1_58–177_ contains a single globular domain spanning residues G75-I171 and comprising six helices: α1 (G75-G85), α2 (K92-E105), α3 (S108-D117), α4 (K124-K140), α5 (K143-R151) and α6 (D155-I171). The N-terminus (S58-L74), which corresponds to the linker to the upstream oligomerization domain of M2-1, is disordered. The α-helix bundle consists of a scaffold, formed by α1, α2, α5 and α6, and an α3–α4 hairpin stacked on α6 (Figure 1A). M2-1_58–177_ displays two oppositely charged faces (Figure 1B). The positively charged face contains a large basic cluster along a grove delimited by helices α2 (K92), α5 (K150, R151) and α6 (K158, K159, K162, K169). Three smaller basic clusters are found on α4 ( K124 and R126), on α3 (K112, K113 and R115), and between α4 and α5 (R139, K140 and K143) as shown in Figure 1B. The putative overall tetrameric domain organization of full-length M2-1 is schemed in Figure 1C.

### RNA binds to the main basic cluster of M2-1_58–177_

Incubation of M2-1_58–177_ with yeast RNA in a ∼1∶1 molar ratio resulted in simultaneous shifting and broadening of several ^1^H-^15^N cross peaks in the ^1^H-^15^N HSQC spectrum of M2-1_58–177_ (see Figure S1). Treatment with RNAse A reversed these effects. This experiment confirmed the RNA binding ability of M2-1_58–177_ *in vitro*. The observed fast to intermediate exchange regime was an indication for a weak interaction. To get rid of the broadening contribution by transversal relaxation of large RNAs twice the molecular weight of M2-1_58–177_, we investigated the RNA:M2-1_58–177_ interaction by NMR by using short synthetic 10–15 nucleotide negative-sense (genomic) RNAs containing selected transcriptional signals [3], [21] as well as their complementary positive-sense sequences. We tested the 3′ polymerase entry site (leader), the U-rich region upstream of the first GS signal, the GS, and the F and SH gene ends (GE_F and GE_SH). Sequences are detailed in Table 1. The oligonucleotides were designed to minimize self-association or formation of secondary structure. Only the leader-neg (5′-CGCAUUUUUUCGCGU-3′) and long U-rich (5′-CCCAUUUUUUUGGUU-3′) sequences were predicted to form hairpins with a poly-U loop and a stem of two or three G-C and A-U pairs with negative free energies. Calculations were carried out on the mfold web server [22]. The absence of self-association was assessed by ^1^H NMR using 200 µM oligonucleotide solutions in water, except for leader-pos and GE_F-pos, for which two broad imino proton signals were observed in the guanosine and uridine regions respectively, corresponding to partial formation of two G-C and U-A base pairs. Finally formation of an RNA duplex with the two complementary strands GE_F-pos and GE_F-neg was observed by NMR with sharp signals corresponding to base-paired uridine imino protons.

**Table 1:** Apparent M2-1_58–177_:oligonucleotide dissociation constants determined from amide (^1^H and ^15^N), methyl (^1^H and ^13^C) and R151-H_δ_ chemical shift variations (at 14.1 T and 293 K).

Designation	Oligonucleotide sequence	K_d_ (µM)^a^
leader-neg	3′-UGCGCUUUUUUACGC-5′	75±15
leader-pos	5′-ACGCGAAAAAAU-3′	2.5±1.5
long U-rich	3′-UUGGUUUUUUUACCC-5′	125±35
short U-rich	3′-GGUUUUUUUA-5′	>600
short A-rich	5′-CCAAAAAAAU-3′	22±6
GS-neg	3′-CCCCGUUUAU-5′	110±15
GE_SH-neg	3′-UCAAUUAAUUUUU-5′	75±15
GE_SH-pos	5′-AGUUAAUUAAAA-3′	13±5
GE_F-neg	3′-UCAAUAUAUUUU-5′	85±25
GE_F-pos	5′-AGUUAUAUAAAA-3′	11±5
GE_F-ds^b^		4.5±2.5
GE_F-DNA	3′-TCAATATATTTT-5′	250±70
UGA2	3′-CGCGAAUUUUUUCGCG-5′	60±10

a K_d_ errors were estimated from standard deviations of K_d_ values determined for individual residues.

b Double-stranded GE_F RNA was formed with equal amounts of the complementary GE_F-neg and GE_F-pos strands.

**Figure 2:**
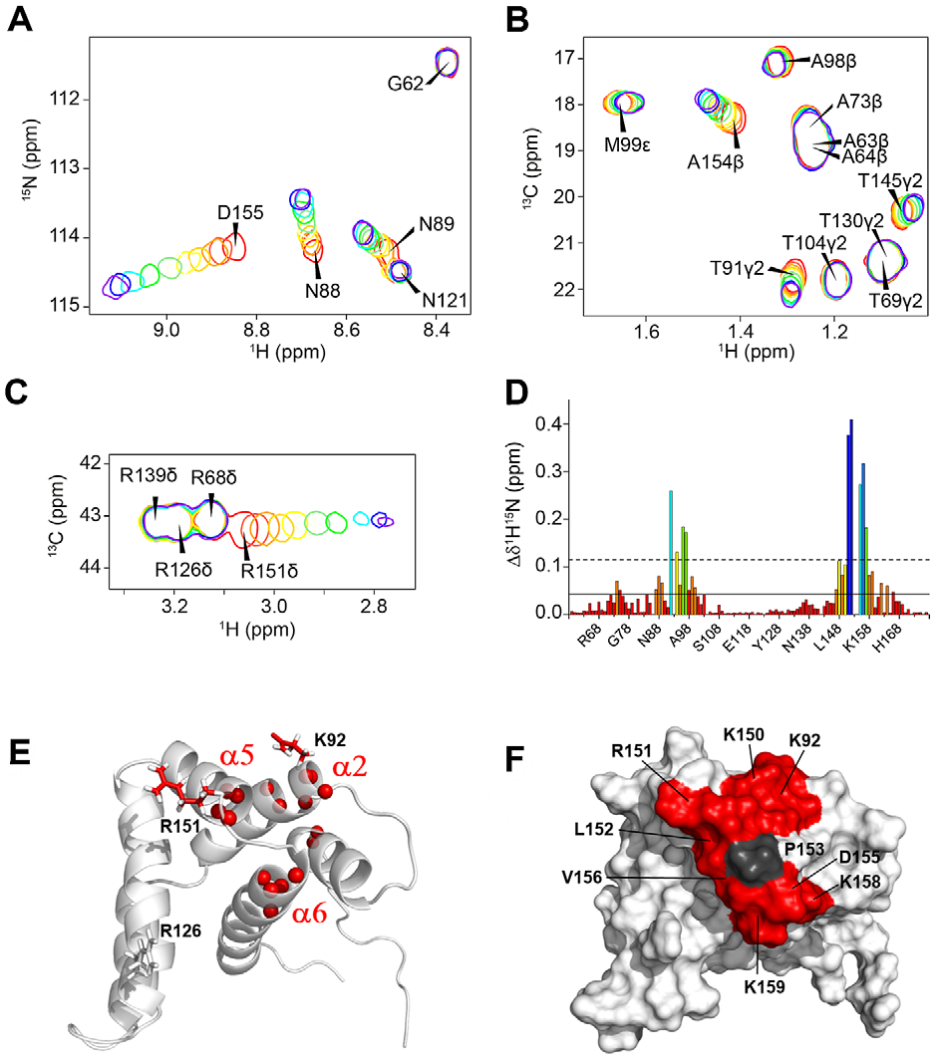
Chemical shift perturbations of M2-1_58–177_ by RNA reveal a continuous RNA binding surface. Panels A, B and C show close-ups of the superposition of HSQC spectra of ^15^N^13^C-labeled M2-1_58–177_ (50 µM, 14.1 T, 293 K) in the presence of increasing amounts of the synthetic short polyA RNA (5′-CCAAAAAAAU-3′). The spectra are shown in colors ranging from red to purple with 0/0.1/0.2/0.4/0.6/0.8/1.0/1.5/2.5/4.0/6.0 RNA equivalents. (A) ^1^H-^15^N HSQC spectrum, (B) methyl region of the ^1^H-^13^C HSQC spectrum and (C) arginine side chain region of ^1^H-^13^C HSQC. (D) Per residue difference plot of weighted averaged chemical shifts Δδ^1^H^15^N of ^15^N-M2-1_58–177_ in the presence of short polyA determined with a 6∶1 RNA∶protein molar ratio. Bars are color coded from red to blue: lowest to highest value. The mean value and mean+1 standard deviation (sd) are indicated with solid and dashed lines. Panels (E) and (F) show the mapping of chemical shift variations (Δδ^1^H^15^N) on the M2-1_58–177_ structure, in cartoon and surface representation respectively. Amide ^15^N atoms of residues with Δδ^1^H^15^N>mean+1sd for all tested RNA sequences (recapitulated in Table 1) are indicated with red spheres. Side chains of R151 and K92, for which RNA induces ^13^Cδ-^1^Hδ and ^13^Cε-^1^Hε chemical shift variations, are in stick representation. The surface formed by all these residues is colored in red. P153, for which no information is available from ^1^H-^15^N HSQC data, is shown in dark grey.

Oligonucleotide binding was followed by chemical shift perturbation experiments in ^1^H-^15^N HSQC and ^1^H-^13^C HSQC experiments, as exemplified for the short polyA sequence in Figure 2 (panels A to C). The chemical shift variation profile in the ^1^H-^15^N HSQC spectrum of short polyA is shown in Figure 2D. Chemical shift perturbation profiles were similar for all RNAs and consistent with those observed for yeast RNA. A complete set of profiles for all oligonucleotides is given in Figure S2. For all RNAs the largest chemical shift changes were observed for residues belonging to the main basic cluster, i.e. K92-V97 (α2), L149-L152 (α5) and D155-K159 (α6), as illustrated in Figure 2E. These residues contribute to forming a continuous positively charged surface, which is consistent with an RNA binding surface (Figure 2F). Chemical shift variations in ^1^H-^13^C HSQC experiments were observed for residues in the same region, solvent exposed methyl side chains in the hinge between helices α5 and α6 as well as R151-H_δ_ protons being affected by RNA binding (see Figure 2B and 2C).

### Short A-rich RSV RNA sequences preferentially bind to M2-1_58–177_

The fast to moderately fast exchange regime in both ^1^H-^15^N and ^1^H-^13^C HSQCs allowed to extract apparent dissociation constants for a binding model with a 1∶1 stoichiometry. The fitted chemical shift variation curves for individual residues in a fast exchange regime are given in Figure S3. The values of the apparent dissociation constants (K_d_) are recapitulated in Table 1. They range from 2.5 µM to >600 µM and fall into two groups. Except for a short U-rich RNA sequence, K_d_s for U-rich negative-sense RNAs are in the 75–125 µM range. UGA2, an RSV-unrelated stem-loop RNA comprising a U-stretch and five base pairs, used as a control, binds with similar affinity (K_d_ = 60 µM). A second control was carried out with a short single stranded DNA equivalent to the GE_F-neg RNA, showing that the same DNA sequence (250 µM) binds with slightly less affinity than the RNA sequence (85 µM). Apparent K_d_s for A-rich positive-sense RNA sequences (leader-pos, GE_SH-pos, GE_F-pos and short A-rich) are in the 2.5–22 µM range. A last experiment with the double-stranded F gene end (GE_F-ds, 4.5 µM) shows that the affinity is further increased as compared to the affinities of single-stranded RNAs. Taken together, these results indicate that M2-1_58–177_ binds to RNA with lower affinity than reported for full-length M2-1 [10], [13]. Importantly they show that the presence of A-rich stretches increases binding affinity as compared to U-rich stretches and that RNA base-pairing might also play a role.

As shown above, helices α2, α5 and α6 form a uniform RNA binding surface. However, residues L74 and G75 in helix α1, which are located on the opposite negatively charged side of the protein, are also affected by RNA binding. For all RNAs, K_d_s determined for residues in helix α1 were always the same as those measured for residues of the main binding site. This strongly suggests that perturbations in α1 on the one hand and in α2, α5 and α6 on the other hand are related to the same binding event. Moreover as the hypothetical second contact surface with RNA would be very distant from the main binding site, we exclude the possibility of a second binding site at α1. However RNA binding to α2 could be transmitted to α1 by slight alterations of helix-helix packing.

### The P binding region of M2-1_58–177_ is adjacent to the RNA binding site

**Figure 3:**
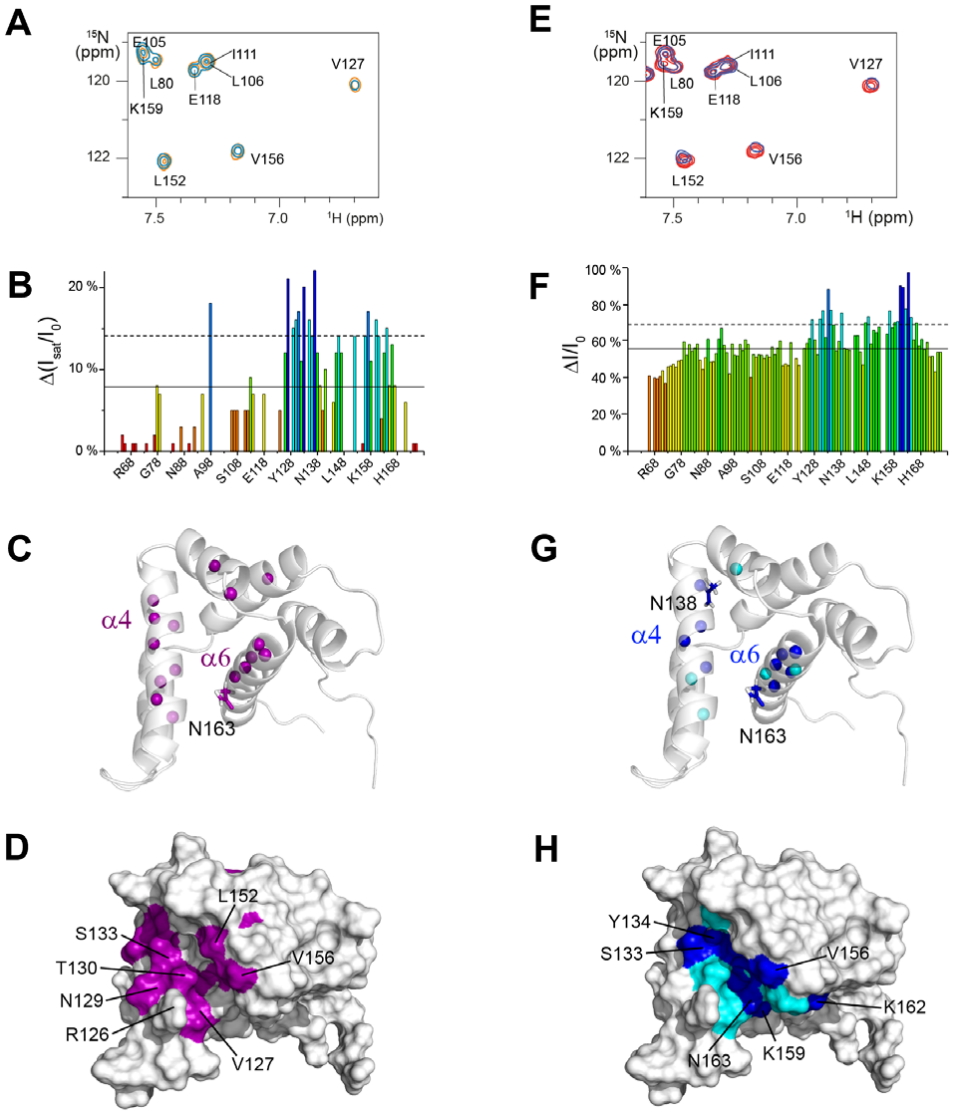
Probing phosphoprotein P binding to M2-1_58–177_ by NMR perturbation experiments. (A, B, C and D) Results of transferred cross-saturation (TCS) experiments carried out with 150 µM ^2^H^15^N-M2-1_58–177_ in the presence of P (15 µM, 91% D_2_O, 14.1 T, 293 K). (A) Close-up of the ^1^H-^15^N HSQC spectra with methyl proton saturation of P (blue) and without (orange). (B) Per residue plot of reduced ^1^H-^15^N HSQC cross-peak intensities, calculated as a ratio between intensities with (I_sat_) and without (I_0_) methyl proton saturation and corrected by the reduced cross-peak intensities measured in the absence of P. (C) and (D) Mapping of the TCS effect on the structure. ^15^N atoms of residues with Δ(I_sat_/I_0_)>mean+1sd are shown as purple spheres. The surface formed by these residues is colored in purple. (E, F, G and H) ^1^H-^15^N HSQC cross-peak intensity perturbation experiments of ^15^N^13^C-M2-1_58–177_ (50 µM) in the presence of P_100–166_ (100 µM, 14.1 T, 298 K). (E) Close-up of the ^1^H-^15^N HSQC spectra with P_100–166_ (blue) and without (red). (F) Per residue plot of the resulting cross-peak intensity reduction relative to the reference intensity. (G) and (H) Mapping of the intensity variations on the structure. ^15^N atoms are shown as spheres for residues with ΔI/I_0_>75% (blue) and >70% (cyan). The same color code is used for the surface representation.

To compare RNA and P affinities for M2-1_58–177_, we performed isothermal titration calorimetry (ITC) experiments using this truncated form of M2-1. Results showed that the phosphoprotein P binds to M2-1_58–177_ with a stoichiometry of 1∶1 and a K_d_ of ∼3 µM (Figure S4). We investigated this interaction further by NMR for atomic details. Perturbations induced by tetrameric full-length P in ^1^H-^15^N and ^1^H-^13^C HSQC spectra of M2-1_58–177_ were monitored. Due to the size of the complex and the unfavorable exchange regime, addition of 0,5 molar equivalents of P was accompanied by extensive overall line broadening in the ^1^H-^15^N HSQC spectra, except for the unstructured N- and C-termini (see Figure S5A). At lower P concentrations, the majority of cross-peaks remained detectable. Residues T130-L165 exhibited larger line broadening, suggesting that P binds to the α4/α5/α6 region. Transferred cross-saturation (TCS) experiments [23] were carried out with ^2^H^15^N-M2-1_58–177_ to more specifically identify residues involved in P binding (Figure 3, panels A to D). Saturation of the methyl protons of P resulted in reduction of M2-1 cross peak intensities in the regions V127-S137 and L152-T164 (helices α4 and α6, and α5/α6 hinge). They form a nearly uniform surface on M2-1 (Figure 3D). Since P residues 100–120 were reported to be critical for M2-1 binding [14], [24], we also monitored intensity variations in ^1^H-^15^N HSQC spectra in the presence of a truncated form of P, P_100–166_, comprising this region as well as the oligomerization domain of P [25], [26]. The results are shown in Figure 3 (panels E to H). Line broadening was enhanced for helices α4 and α6 (Figures 3F and 3G), which is consistent with the results obtained with full-length P. In summary, P binds to a region proximal to the RNA anchoring surface. Helices α6 and α5 were sensitive to both P and RNA binding in our NMR experiments, and could thus contribute to both interaction sites, while α4 and α2 appear to be involved specifically in P binding and RNA binding, respectively.

### Effects of surface mutations in the P and RNA binding regions on M2-1 controlled transcription probed by a minigenome

**Figure 4:**
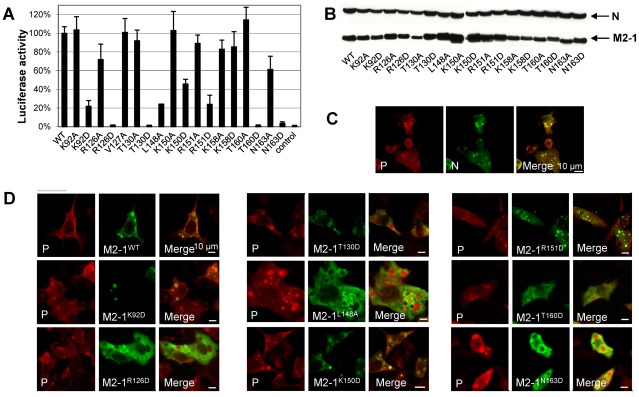
Effect of M2-1 mutations on RSV transcription and association with the nucleocapsid. (A) Analysis of RSV specific M2-1-controlled transcription with WT and M2-1 substitution mutants. BSRT7/5 cells were transfected with RSV pP, pN, pL, and pM2-1 plasmids and an RSV specific minigenome containing the firefly luciferase reporter gene, together with p-β-Gal constitutively expressing β-galactosidase. Luciferase activity, measured 24 h after transfection, was normalized by β-galactosidase activity, and the luciferase activity gained with WT M2-1 set to 100%. The mean value and confidence intervals (error bars) result from 3 separate experiments performed in duplicate. A control was run without M2-1. (B) Expression of M2-1 mutant proteins in BSRT7 cells. Cells were co-transfected with plasmids encoding M2-1 mutants and N. Cell extracts were analyzed by Western blotting with rabbit polyclonal antibodies against M2-1 and N. Expression levels of M2-1 were normalized against N expression and compared to tubulin. (C, D) Colocalization studies of M2-1 with N-P complexes. Plasmids encoding N, P, and M2-1 mutants were transfected into BSRT7/5 cells. Immunofluorescence analysis was performed on cells fixed 24 h after transfection, by using rabbit polyclonal anti-N (1∶100) or anti-P (1∶500) and Alexa Fluor 594 goat anti-rabbit (1∶1000) antibodies, and mouse monoclonal anti-M2-1 (1∶40 dilution) and Alexa Fluor 488 goat anti-mouse (1∶1000) antibodies. Horizontal bars correspond to 10 µm.

Based on these results, we designed single residue mutations of the full-length M2-1 protein targeting the binding sites of P and RNA. Solvent-exposed residues were first substituted by Ala. The effect of these mutations on transcription antitermination by M2-1, which was previously shown to be the same for RSV-specific and heterologous sequences [6], was assessed using an RSV dicistronic subgenomic replicon, pM/Luc. It contains the authentic M/SH gene junction, and the Luc reporter gene downstream of the gene start sequence present in this gene junction. The expression of the Luc gene in this system is absolutely dependent on the presence of a functional M2-1 [15]. The pM/Luc plasmid was co-transfected in BHK-21 BSRT7/5 cells expressing T7 RNA polymerase together with p-β-gal, pL, pP, PN, and pM2-1. Luciferase activities were determined and normalized based on β-galactosidase expression [5], [8], [15], [27], [28]. Except for L148A and N163A, most of the Ala substitutions had only little effect on transcription, as assessed by Luc expression (Figure 4A). As the P- and RNA-binding surface identified by NMR is highly positively charged, residues were substituted by Asp to emphasize the electrostatic effect of mutations. When substituted by Asp, K92 (in helix α2), a residue involved in RNA but not in P binding according to the NMR results, and R126 and T130 (in helix α4), two residues involved in P but not in RNA binding, appeared to be critical for transcription. A similar effect on transcription was observed for four other residues (K150, R151, T160 and N163, in helices α5 and α6), that belong to the dual RNA/P binding surface. In contrast, Ala and Asp mutants of K158, which is also located in the RNA/P binding region, still displayed more than 80% activity as compared to the WT (Figure 4A). As a control, we further verified by NMR that the deactivating mutations did not disrupt the protein fold (Figure S6).

### Interaction of M2-1 surface mutants with P-N complexes *in cellulo*

Co-expression of P and N proteins (in the absence of other viral proteins) in cells induces the formation of cytoplasmic inclusion bodies containing P-N complexes, as observed in RSV infected cells [11]. When co-expressed with P and N, WT M2-1 also localizes preferentially in these IBs as seen in Figure 4D [11]. We analyzed the intracellular localization of the M2-1 mutants in cells co-transfected with expression vectors for P, N and M2-1 by fluorescence microscopy (Figure 4C and 4D). Contrary to WT M2-1, the mutants R126D, T130D, L148A, T160D and N163D were excluded from the IBs and spread all throughout the cytoplasm. In contrast K92D, K150D and R151D retained their localization to cytoplasmic IBs. These mutants were expressed at comparable levels, as determined by Western blotting (Figure 4B), and were correctly folded when purified from *E. coli* and analyzed by NMR (Figure S6).

### *In vitro* binding of M2-1 mutants to P and RNA

**Figure 5:**
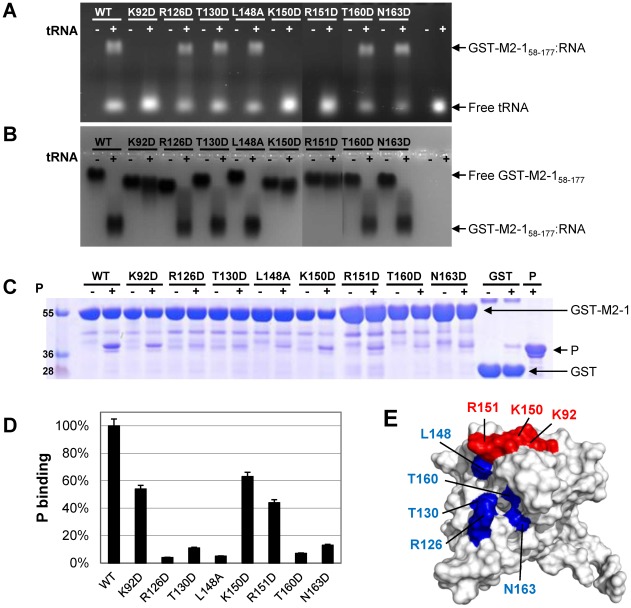
Effect of mutations affecting M2-1-controlled transcription on M2-1_58–177_:RNA and M2-1:P complex formation *in vitro*. (A and B) Electrophoretic mobility shift assay (EMSA) of M2-1:RNA complex formation. Eluted GST-M2-1_58–177_ (WT and mutants selected using the minigenome assay, 100 µM final concentration) were incubated with yeast tRNA (∼50 µM final concentration) for 1 h at room temperature. (A) Complexes were resolved by agarose gel electrophoresis stained with ethidium bromide. (B) Proteins were revealed by amido black staining. M2-1 mutations are indicated above each lane. (C and D) GST pull-down of purified P by GST-M2-1 (WT and the same mutants as in A and B). (C) GST-M2-1 or GST were incubated alone (−) or in the presence of P (+), washed, and analyzed by SDS-PAGE. P was also run alone (lane P). (D) Coomassie blue-stained gels were scanned and M2-1:P binding was quantified using ImageJ software and corrected for nonspecific binding to GST. Errors were estimated to ±5%. (E) The surfaces formed by the 8 residues, for which mutants were analyzed, are indicated on the M2-1 structure according to their binding partner: in red (RNA binding) and blue (P binding).

To verify that the residues identified using NMR and the minigenome assay are critical for RNA- and/or P-binding, the *in vitro* RNA and P binding capacities of eight M2-1 mutants selected by the Luc assay were investigated. As M2-1 did not migrate in native agarose gel, it was not possible to obtain electrophoretic mobility shift assays (EMSA) with the GST-free forms. We thus used M2-1 fused to GST for the *in vitro* binding assays with RNA and P. For RNA binding assays, we used either full-length (tetrameric) or truncated 58–177 (monomeric) forms of M2-1 fused to GST. GST-M2-1_58–177_, incubated with tRNA, was analyzed by EMSA. Formation of GST-M2-1_58–177_:RNA complexes was only impaired by the K92D, K150D and R151D mutations, which did not prevent M2-1 association with IBs (Figure 5A and 5B). The effect of single mutations of the full-length form on *in vitro* RNA-binding was smaller than with truncated M2-1_58–177_, probably owing to the higher avidity of tetramers for RNA compared to monomers, and the Lys/Arg repetitions that limit the effect of single substitutions (data not shown). *In vitro* M2-1:P interactions were assessed by GST pull-down assays. As shown in Figure 5C and 5D, a≥50% decrease in P binding was observed for the mutants R126D, T130D, L148A, T160D and N163D, which were excluded from IBs, but could still bind RNA. The two separate binding surfaces for RNA and P, determined from these 8 residues, are illustrated in Figure 5E. Together with the cellular localization experiments, these results show that there is a strong correlation between the reduced capacity of M2-1 to pull-down P *in vitro* and its exclusion from IBs. Conversely, since residues specifically involved in RNA binding appear not to be crucial for recruitment of M2-1 to IBs, the M2-1:RNA interaction is not required for this process.

## Discussion

### Similarity between pneumovirus M2-1 and filovirus VP30 transcription co-factors

In contrast to other members of the *Mononegavirales* order for which transcription is driven by only three proteins (N/NP, P and L), pneumoviruses and filoviruses encode a fourth transcription co-factor (M2-1 and VP30 respectively). Parallels have been drawn between VP30 and M2-1 on the basis of their functions and domain organization [17], [29]. Both were shown to be necessary for efficient transcription in reconstituted minigenome systems [7], [30]. In addition, although VP30 and M2-1 were shown to be dispensable for RNA replication [30], [31], [32], both are required to rescue recombinant EBOV or Marburg virus and RSV [33], [34], [35]. M2-1 and VP30 contain an N-terminal Cys_3_His_1_ motif that does not bind RNA directly, but that is essential for VP30 RNA binding [15], [17], [36], [37]. In the case of M2-1 this motif is indispensable for function, but its exact role is still unknown [17], [18], [19]. Like M2-1, VP30 also contains an oligomerization domain downstream of the Cys_3_His_1_ motif, which is necessary for its function during transcription [15], [36]. But, despite functional similarities, the sequence identity between the two proteins is very low and it was not possible to know that they were evolutionary related.

**Figure 6:**
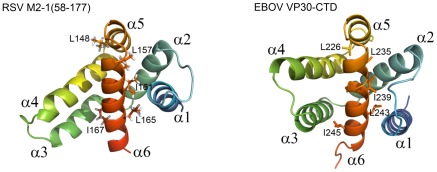
Structural alignment of RSV M2-1_58–177_ and EBOV VP30_CTD_. Cartoon representations of aligned RSV M2-1_58–177_ (residues L74 to T172) and EBOV VP30_CTD_ (pdb 2I8B, [38]). Disordered N- and C-termini are not shown. Structural alignment with M2-1_58–177_ was generated by the Dali server [39] (Z-score = 5.7; rmsd = 3.9 Å; 92 aligned residues; 9% sequence identity). VP30_CTD_ helix α7, which has no counterpart in M2-1_58–177_, is not represented. Identical hydrophobic residues in helices α5 and α6 are represented with sticks for M2-1_58–177_ and VP30_CTD_.

Here we show that the globular domains M2-1_58–177_ and EBOV VP30_CTD_ [38] are structurally homologous and that they display the same α-helix bundle fold. The structures were aligned on the DALI server [39] which yielded a Z-score of 5.7 and an rmsd of 3.9 Å for 92 aligned residues and 9% sequence identity (see Figure 6). The core helices α1, α2, α5 and α6 align well, with a slight shift of α1_M2-1_ with respect to α1_VP30_. The α3–α4 hairpins are skewed relative to each other. Moreover M2-1_58–177_ and VP30_CTD_ contain several identical hydrophobic residues in helices α5 and α6 (indicated in Figure 6), which are involved in stabilizing inter-helical contacts and appear to be semi-conserved among pneumovirus/metapneumovirus M2-1 and filovirus VP30 protein sequences (Figure S7). Although they mainly contribute to inter-helix packing, it is noteworthy that one of them (L148) is also critical for M2-1 transcription antitermination. VP30_CTD_ contains an additional 7th C-terminal helix, which has no counterpart in M2-1. It stabilizes the crystallographic VP30_CTD_ dimer by interaction with α1. Since full-length M2-1 forms tetramers with the oligomerization domain just upstream of the core domain, its monomeric state in isolation suggests that the core domains may at best be loosely associated with each other in the tetramer, in the absence of partner molecules, as schemed in Figure 1C.

The overall structural match rationalizes the relationship between M2-1 and VP30. Importantly both proteins associate with the nucleocapsid by means of their globular core domains [10], [14], [38], [40]. Whereas the VP30_CTD_:nucleocapsid interaction is mediated by the EBOV nucleoprotein but not by RNA [41], the M2-1:nucleocapsid interaction has been proposed to be mediated by RNA [10] and by P [14]. RNA binding to VP30_CTD_ has not been observed [38], in contrast to what we report for M2-1_58–177_. As no direct M2-1:N complex was evidenced *in vitro* [10], [14], the correlation found between M2-1 localization to cytoplasmic IBs, containing N and P, and the capacity of M2-1 to bind P *in vitro* indicates that binding of M2-1 to P drives recruitment of M2-1 to the holonucleocapsid.

### M2-1_58–177_ binds to mRNA transcripts rather than to genomic RNA

M2-1 was first described as an antitermination factor, preventing cessation of chain elongation and release of the nascent mRNA [7]. Later M2-1 was reported to inhibit transcription termination at the GE signals to produce polycistronic readthrough mRNAs [6], [8], [9], depending on GE sequences. The semi-conserved 12–13 nucleotide GEs fall into three groups with respect to this property: the first (NS1/NS2, NS2/N, M2/L, and L/trailer) contains sequences inefficient for transcription termination and is insensitive to M2-1; the second (N/P, P/M, M/SH, SH/G, G/F) is very efficient in transcription termination but not sensitive to M2-1; finally (F/M2) is highly sensitive to M2-1 [9]. M2-1 did not direct readthrough at the leader-NS1 junction [6]. Thus, it was suggested that M2-1 would not only prevent inappropriate intragenic termination, but also allow the polymerase to access to promoter-distal regions of the genome and to transcribe downstream genes [9]. Cuesta et al. reported that renatured M2-1 bound preferentially to a short positive-sense leader RNA *in vitro* [13], while Cartee and Wertz co-immunoprecipitated RSV mRNA with M2-1 from infected cells treated with Actinomycin D [10]. These observations suggested that M2-1 could modulate transcription termination by recognizing specific viral RNA sequences, either on transcribed mRNAs or on the genomic RNA template. Contrary to transcribed viral mRNA, genomic RNA is encapsidated. However if the model of RNA synthesis by the vesicular stomatitis virus, a prototype of nonsegmented negative-strand RNA viruses, could be transposed to RSV, genomic RNA would become accessible as the nucleocapsid is being locally disassembled by the transcription complex [42], [43]. By comparing the affinities of M2-1_58–177_ for short 10–15 nucleotide RSV RNA sequences, we found that M2-1_58–177_ bound preferentially to positive-sense RNA sequences such as the 12 first bases of the positive-sense leader (K_d_ = 2.5 µM), positive-sense GEs (11 and 13 µM) and an A-rich sequence located on the positive-sense leader (22 µM). Affinities of the negative-sense signal sequences were systematically lower by one order of magnitude or more than those of their complementary sequences. But there was no difference between the SH and F gene ends, which are respectively insensitive and highly sensitive to M2-1. The 3′ and 5′ extremities of the leader and the GS displayed a similar behavior to that of GEs of same polarity, indicating that M2-1_58–177_ does not discriminate between transcription signals of same polarity. Sequence specificity of RSV transcription antitermination thus appears not to be linked to the M2-1 core domain. These results are also consistent with the observation that M2-1 is not required for initiation of RNA transcription [7]. Our results show that M2-1_58–177_ displays a preference for purine-rich and especially A-rich RNAs found in positive-sense RSV RNAs over pyrimidine-rich sequences containing U-stretches found in negative-sense RNAs. Thus, they reinforce the hypothesis that M2-1 binds preferentially to positive sense RNA transcripts rather than to the template, in agreement with previous reports [10], [13]. An additional finding is that M2-1 binds to the double-stranded F gene end with similar or better affinity (4.5 µM) than to the positive-sense sequence alone (11 µM). This implies that M2-1 could bind to the nascent mRNA transcript, either still bound to the template or released from it. In both cases, in order to facilitate transcription elongation, M2-1 could prevent formation of mRNA secondary structures that might destabilize the transcription complex, in analogy to the function of N protein that binds to the nascent RNA during replication.

### Functional implications of the interaction of M2-1 with P

Using *in vitro* experiments, we have previously shown that RNA and P bind to M2-1_58–177_ in a competitive way [15]. Here we have characterized two distinct binding surfaces for P and RNA by NMR (Figure 5E). Their edges are partially overlapping. The P binding epitope, which is buried between helix α4 and the hinge between α5 and α6 (Figure 3D and 3H), might be occluded by RNA binding in the vicinity, and conversely RNA binding might be hampered by bound P due to steric hindrance. Both binding sites are located on the positively charged face of M2-1_58–177_, underlining the importance of electrostatic interactions for association with RNA and with P. This hypothesis is also in agreement with the predicted negatively charged surface in computer models of the M2-1 binding region of the P tetramer, spanning residues 100–120 of P [44]. Redundancy of positive charges in the P and RNA binding region certainly accounts for the weak effect on Luc expression observed for Ala mutants of residues in this region in the minigenome assay, as compared to Asp substitution, which introduced opposite charges. Electrostatic interactions could be further emphasized in the M2-1 tetramer if the core domains were arranged to provide an extended binding surface consisting of two adjacent positively charged clusters. In addition to charge effects, specific hydrophobic interactions may also contribute, as suggested for the interaction between P and M2-1 in [14]. This could apply to RNA as well. Although the range of RNA affinities for M2-1_58–177_ (micromolar to millimolar) is an indicator for non-specific binding mediated by the RNA phosphate backbone, corroborated by binding of a DNA sequence equivalent to GE_F-neg, the difference between A-rich and U-rich RNA affinities indicates that the nature of the bases also comes into play.

The large M2-1_58–177_:RNA binding surface determined by NMR coincides with the main positively charged cluster of M2-1_58–177_ (Figure 1B and 2F), which is well conserved among *Pneumovirinae* (Figure S7). We confirmed by mutagenesis that the three basic residues K92, K150 and R151, included in this epitope, are crucial for *in vitro* RNA binding to M2-1_58–177_ and for transcription enhancement *in vivo* by full-length M2-1. However they did not prevent association with cytoplasmic IBs or P binding. In contrast, mutants R126D, T130D, L148A, T160D and N163D, for which M2-1-controlled transcription was impaired and which still bound RNA *in vitro*, had lost their ability to bind P *in vitro* and did not co-localize with N-P complexes in cytoplasmic IBs. These residues are not well conserved in *Pneumovirinae* M2-1 proteins (Figure S7A), but this would be consistent with the sequence variability of the M2-1 binding region of P(100–120), which has co-evolved with M2-1. Altogether the NMR and mutagenesis results provide a rationale for the competitive binding previously observed between P and RNA [15]. They highlight the role of P for the recruitment of M2-1 to cytoplasmic IBs that contain N and P and where viral RNA synthesis takes place, by analogy to *Rhabdoviridae* [12].

Possible cooperative effects in full-length tetrameric M2-1, involving the N-terminal Cys_3_His motif and interactions between core domains, may induce an increased affinity for both P and RNA as well as sequence specific recognition of RNA, with respect to the core domain of M2-1. However the overall lower affinities of RNA for M2-1_58–177_, as compared to P, suggest that M2-1 binds P preferentially in the absence of viral RNA so that M2-1 would be recruited as an RNA-free M2-1:P complex to the IBs. This hypothesis is in agreement with our fluorescence microscopy observations, since WT M2-1 and mutants that do not bind RNA are found likewise in IBs containing N and P (Figure 4C and 4D). Indeed, higher affinity to RNA would result in sequestration of M2-1 in the cytoplasm, where cellular RNA is highly accessible, and M2-1 would not be recruited to the IBs. The relatively low K_d_s are in the same range as those measured for the Measles virus P-N interaction [45], this intrinsically weak association being probably required for movement of the viral polymerase during RNA transcription.

In summary, our results indicate that not only P:M2-1, but also RNA:M2-1 interactions are required for efficient transcription activation by M2-1. Association with P is strictly required for recruitment to the viral RNA synthesis site. As suggested by the proximity of the binding sites, it is likely that the P:M2-1 interaction is displaced in favor of other interactions, RNA:M2-1 interactions in particular, in the context of the holonucleocapsid. The higher affinity of M2-1_58–177_ for the 5′ end of the positive-sense Leader RNA (2.5 µM), which is in the same range as the affinity for P (3 µM), suggests that M2-1 could be loaded onto the polymerase during transcription initiation. It can further be speculated that an L:M2-1 interaction also takes place when the P:M2-1 interaction breaks down, thus altering the sensitivity of the polymerase to transcription termination signals. A similar L:VP30 interaction was recently reported for EBOV [46]. This hypothesis is currently under investigation for RSV.

## Materials and Methods

### Protein expression and purification

Recombinant P, P_100–166_, M2-1 and M2-1_58–177_ (WT and mutants) as well as ^15^N-,^13^C- and/or ^2^H-labeled M2-1_58–177_ were expressed in *E. coli* BL21(DE3) strain. Full-length M2-1 and M2-1_58–177_ were produced as GST fusion proteins as described in [15] and [25] with a thrombin cleavage site. M2-1 amino acid substitution mutants were obtained with the Quickchange site-directed mutagenesis kit (Stratagene) by using pGEX-M2-1, pGEX-M2-1_58–177_ and pM2-1 as templates. Isotopically labeled proteins for NMR were produced in minimal M9 medium supplemented with 1 g/L ^15^NH_4_Cl and 4 g/L ^13^C- or unlabeled glucose (Cortecnet). Protocols are detailed as Supplemental Materials and Methods (Text S1).

### Minigenome assay

BSRT7/5 cells stably expressing T7 RNA polymerase [47] were maintained in EMEM supplemented with 10% FCS/l-glutamine/penicillin-streptomycin solution. The cells were grown in an incubator at 37°C under 5% CO_2_. pN, pP, pM2-1 and pL plasmids coding for HRSV (strain Long) N, P, M2-1 and L proteins respectively, under the control of the T7 promoter, have been described previously [15], [48]. An encephalomyocarditis virus internal ribosome entry site (IRES) sequence was placed between the T7 promoter and the inserted ORF to enhance protein expression in BSR/T7-5 cells, as previously described [48], [49]. The pM/Luc subgenomic replicon was derived from the pM/SH subgenomic replicon [8] and has been described previously [15]. It contains two transcription units, the second encoding the firefly luciferase (Luc) gene under the control of the M/SH intragenic sequence. The expression of the Luc gene in this system is absolutely dependent on the presence of M2-1 [15]. BSRT7/5 cells were transfected with pN, pP, pL, pM2-1, p-β-Gal coding for beta-galactosidase under the control of the Rous sarcoma virus promoter (Promega) and pM/Luc, where luciferase expression is controlled by the RSV M/SH intergenic region [15]. Luciferase activity was determined in triplicate 24 h post-transfection as previously described [15]. Cells were lysed in luciferase lysis buffer (30 mM Tris, pH 7.9, 10 mM MgCl_2_, 1 mM dithiothreitol [DTT], 1% [vol/vol] Triton X-100, and 15% [vol/vol] glycerol), and luciferase activities were evaluated twice for each cell lysate with an Anthos Lucy 3 luminometer (Bio Advance).

### Transfections and indirect immunofluorescence analysis

BSRT7/5 cells were transfected with pP (0.4 µg), pN (0.4 µg) and pM2-1 (0.2 µg) containing either WT or mutant M2-1 by using Lipofectamine2000 (Invitrogen). Samples were fixed after 24 h in 4% paraformaldehyde, and permeabilized in PBS containing 0.1% Triton X-100 and 3% BSA. Each coverslip was incubated with primary antibodies: anti-N (1∶100 dilution) and anti-P (1∶500 dilution) rabbit polyclonal sera, and 37M2 and 22K4 anti-M2-1 monoclonal antibodies (1∶40 dilution) [50]. These samples were incubated for 1 h at room temperature, washed, and then incubated for an additional hour with Alexa Fluor 488 goat anti-mouse and Alexa Fluor 594 goat anti-rabbit (1∶1000) IgG (Invitrogen). Cells were observed with a Nikon TE200 inverted microscope equipped with a Photometrics CoolSNAP ES2 camera. Images were processed using MetaVue software (Molecular Devices).

### Analysis of *in vitro* RNA and P binding by M2-1 and M2-1 mutants

GST-M2-1_58–177_ fusion proteins (WT or mutants, final concentration 100 µM) were eluted by GSH and incubated with yeast tRNA (Sigma, final concentration 50 µM) in a final volume of 10 µl. Complexes were resolved by 1.5% agarose gel electrophoresis in 1× Tris-Glycine buffer at 4°C, stained with ethidium bromide and amido black. GST pull-down of purified recombinant P by full-length GST-M2-1 fusion proteins (WT and mutants) was performed by incubating 10 µl aliquots of a 50% slurry of Glutathione-Sepharose 4B beads (GE Healthcare) containing ∼25 µM GST-M2-1 in PBS with a 3-fold molar excess of P for 1 h at 20°C under agitation. Beads were washed extensively with PBS, boiled in 25 µl Laemmli buffer and analyzed by SDS-PAGE and Coomassie blue staining. Bands were quantified using ImageJ software. Affinity between P and M2-1 was determined by isothermal titration calorimetry.

### Isothermal titration calorimetry assays

Raw ITC data were processed and quantitative analysis of the P-M2-1_58–177_ interaction was performed on a MicroCal ITC200 microcalorimeter (Microcal, Northampton, MA). Samples were dialyzed against 1× PBS for 15 h. The experiments were carried out at 20°C. The P concentration in the microcalorimeter cell (1.4 mL) was of 33 µM. In total, 20 injections of 2 µL of M2-1_58–177_ solution (concentration 335 µM) were carried out at 180 s intervals, with stirring at 1000 rpm. The experimental data were fitted to a theoretical titration curve with software supplied by MicroCal (ORIGIN). This software generates titration curves based on the relationship between the heat generated by each injection and ΔH (enthalpy change in kcal/mol), K_d_ (dissociation constant), n (number of binding sites), the total protein concentration and free and total ligand concentrations.

### RNA oligonucleotide synthesis

10–15 nucleotide RNAs corresponding to viral RNA sequences of negative and positive polarity were synthesized on a Pharmacia Gene Assembler Plus using phenoxyacetyl β-RNA phosphoramidites and Universal Support resin (Glen Research) with a protocol adapted for RNA from DNA solid-phase synthesis [51]. The products were HPLC-purified (Beckman) by anionic chromatography on a DEAE-Sepharose column (Pharmacia) in 10 mM phosphate buffer pH 6.8 and eluated with a linear gradient from 0.55 to 1 M NaCl in 80 min. The oligonucleotides were extensively dialyzed in water. If necessary the pH was adjusted to 7 with 0.5 M NaOH and the concentration determined using the A260 value. Aliquots were then lyophilized and stored at −20°C. The oligonucleotide sequences are given in Table 1. Folding properties of RNAs were estimated by using the mfold web server [22].

### NMR spectroscopy

NMR measurements were carried out at 293 K (or 298 K) on Bruker Avance 600, 800 and 950 MHz spectrometers equipped with triple resonance cryoprobes. All samples were in 50 mM sodium phosphate buffer pH 6.8, 150 mM NaCl, 1 mM DTT and 7% D_2_O. Resonance assignment was reported elsewhere [52]. ^15^N-NOESY-HSQC (800 MHz) and ^13^C-NOESY-HSQC (950 MHz) experiments, simultaneously edited for aliphatic and aromatic ^13^C, with 80 and 100 ms mixing times, were recorded using 120 µM ^15^N- M2-1_58–177_ and 100 µM ^13^C-M2-1_58–177_ samples respectively. Data were processed with Topspin 2.1 or NMRPipe [53]. Spectra were analyzed with Sparky [54]. Residual dipolar couplings (RDCs) were collected on ^15^N-labeled M2-1_58–177_ in two alignment media. The first sample contained 120 µM ^15^N-M2-1_58–177_ in a stretched 6% acrylamide/bisacrylamide (37.5∶1) gel (deuterium splitting Δ^2^H = 3 Hz). The second sample contained 200 µM ^15^N-M2-1_58–177_ in a 5% hexanol/C12E5 phase with r = 0.96 molar ratio (Δ^2^H = 26,7 Hz). ^1^D_NH_ RDCs were extracted from spin-state selective InPhase-AntiPhase ^1^H-^15^N HSQC experiments acquired in interleaved fashion.

### NMR structure calculation

M2-1_58–177_ structures were calculated with CYANA 2.1 [55], using distance restraints obtained from ^15^N- and ^13^C-NOESY-HSQC spectra and backbone torsion angles generated with TALOS. They were refined in Xplor-NIH [56] using ^1^D_HN_ residual dipolar couplings measured in a stretched polyacrylamide gel and a hexanol/C12E5 phase. Structure statistics are summarized in Table S1. More details are provided in Text S1. Graphic representations were performed with PyMOL [57].

### NMR M2-1_58–177_ chemical shift and cross-peak intensity variation experiments with RNA and phosphoprotein

Chemical shift and cross-peak intensity perturbations experiments were carried out with yeast RNA (Roche) and short synthesized oligonucleotides (see Table 1). 0.25–20 equivalents of lyophilized RNA were added stepwise to 50–60 µM ^15^N-,^13^C- or ^15^N^13^C-labeled M2-1_58–177_. ^1^H-^15^N and ^1^H-^13^C HSQC spectra were recorded at 293 K at each step, at magnetic fields of 14.1 T and/or 22.3 T.

Interaction with phosphoprotein was probed by cross-peak intensity perturbations in ^1^H-^15^N and ^1^H-^13^C HSQC experiments, with full-length P (P_1–241_) and truncated P_100–166_. Samples with P_1–241_ were prepared by mixing concentrated solutions of P_1–241_ (30 µM) and ^15^N^13^C-labeled M2-1_58–177_ (275 µM) in the same phosphate buffer to a final concentration of 40 µM M2-1_58–177_ and P∶M2-1 molar ratios of 0.25∶1:and 0.5∶1. Samples with P_100–166_ were prepared by mixing solutions of P_100–166_ (600 µM) and ^15^N^13^C-labeled M2-1_58–177_ (140 µM) to a final concentration of 45 µM M2-1_58–177_ and a P∶M2-1 molar ratio of 2∶1.

### Titration experiments with RNA and dissociation constant determination

Spectra were typically recorded at 14.1 T and 293 K. For each RNA sequence, addition of RNA was followed by ^1^H-^15^N HSQC experiments of ^15^N-M2-1_58–177_. Weighted averaged chemical shift differences were calculated from ^1^H and ^15^N chemical shifts according to following equation, where the 1/10 scaling factor for ^15^N chemical shifts corresponds to the ratio of gyromagnetic ratios of ^15^N and ^1^H: 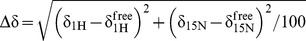.

The apparent dissociation constant K_d_ was obtained by fitting ^1^H or ^15^N chemical shift differences at each titration point with two parameters in a binding model with 1∶1 stoichiometry and with a user-defined function in Origin 7 software as follows: 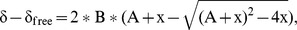
where 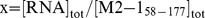, 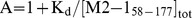, 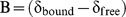.

### Transferred cross-saturation experiments

Samples were prepared with a 30 µM solution of P_1–241_ centrifuged at 15 krpm in a TLA55 rotor (Beckman) for 15 min and exchanged into D_2_O buffer with biospin columns according to the manufacturer's instructions (Biorad). Transferred cross-saturation experiments [23] were conducted with 150 µM ^2^H^15^N labeled M2-1_58–177_ in 91% D_2_O in the presence of 15 µM (10%) unlabeled P_1–241_ protein. Broadband proton saturation (2 s) was achieved with a 1.8 kHz Wurst pulse centered at 0.8 ppm. Spectra were recorded in interleaved fashion with and without saturation, with 1.5 s recycling delay, at 14.1 T and 293 K. Intensity ratios were determined based on experiments with and without saturation. Control experiments were carried out without P to account for spin diffusion and effects of residual aliphatic protons in M2-1_58–177_.

### Data deposition

Atomic coordinates and structural constraints have been deposited in the Protein Data Bank (PDB accession code 2L9J).

### Accession numbers

Swiss-Prot P04545.1

UniProt P28887.1

## Supporting Information

Figure S1 **Binding of yeast RNA to ^15^N-M2-1_58–177_ followed by ^1^H-^15^N HSQC spectra.** Measurements were done at 298K and a field of 14.1 T. The reference spectrum of ^15^N-M2-1_58–177_ (50 µM) is shown in black, the spectrum after addition of 4 mg/mL yeast RNA (∼1∶1 molar ratio) in red and after treatment by RNAse A in green.
(EPS)

Figure S2 **^1^H-^15^N chemical shift perturbations measured for ^15^N-M2-1_58–177_ in the presence of RNA.** ^1^H-^15^N HSQC spectra were recorded at 14.1 T and 293 K with 50 or 60 µM protein. Per-residue plots of combined ^1^H/^15^N chemical shifts in absolute values are represented for (a) Yeast RNA (Roche) (4 mg/mL), for (b) an RSV unrelated hairpin UGA2 hairpin (20∶1 RNA∶protein molar ratio), for single-stranded RSV genomic negative-sense (neg) and positive-sense (pos) RNA sequences, (c) neg leader (6∶1), (d) neg gene start (2.5∶1), (e) neg SH gene end (2.5∶1), (f) neg F gene end (2.5∶1), (g) neg long U-rich (30∶1), (h) neg short U-rich (7.5∶1), (i) pos short A-rich (6∶1), (j) pos leader (2∶1), (k) pos SH gene end (6∶1), (l) pos F gene end (6∶1) and for (m) double-stranded F gene end (6∶1) and (n) a DNA equivalent to neg gene end F (4∶1). The exact sequences are given in Table 1. Mean and mean+1sd values are indicated by solid and dashed lines respectively.
(EPS)

Figure S3 **Determination of apparent RNA dissociations constants by titration experiments with ^1^H-^15^N HSQC experiments of ^15^N-M2-1_58–177_.** ^1^H and ^15^N chemical shift differences of backbone amides or Q93 side chain (denoted H, N, Hε21, Hε22, and Nε2 respectively) are displayed versus the ratio of RNA concentration relative to protein for chosen residues showing the largest chemical shift amplitudes. Measurements were done at 14.1 T and 293 K. Protein concentration was 50 or 60 µM. ^15^N chemical shifts were scaled with a factor 1/10 to be of comparable magnitude to ^1^H. Plots are shown for following RNA sequences, “pos” and “neg” denoting RSV genomic positive-sense and negative-sense sequences : (a) UGA2 hairpin, (b) neg leader, (c) neg gene start, (d) neg SH gene end, (e) neg F gene end, (f) neg long U-rich, (g) pos SH gene end, (h) pos short A-rich, (i) pos F gene end and (j) double-stranded F gene end. The data were fitted to a binding model with 1∶1 stoichiometry in Origin software. The mean values of apparent K_d_ and the statistical error are indicated for each RNA.
(EPS)

Figure S4 **ITC binding isotherms for P binding to M2-1_58–177_.** (A) Raw binding data obtained for 20 automatic injections of M2-1_58–177_ (2 µl for each injection, 335 µM protein concentration) into a cell containing P (200 µL initial volume; 33 µM initial protein concentration). Proteins were suspended in 1× PBS. (B) Integrated titration curve obtained from the raw data in panel A after baseline subtraction. The solid squares represent the experimental data, while the solid line corresponds to the standard multiple independent binding-site model that was fitted to the data. The corresponding average K_d_ value is 3 µM with a stoichiometry of 1∶1.
(TIF)

Figure S5 **Intensity variations of M2-1_58–177_ HSQC spectra in the presence of phosphoprotein.** (A) Superposition of ^1^H-^15^N HSQC spectra of ^15^N^13^C-labeled M2-1_58–177_ without (black) and with 0.5 equivalent of full-length RSV phosphoprotein P (blue) shows overall line broadening on addition of P. (B) Arginine ^13^C_δ_-^1^H_δ_ and lysine ^13^C_ε_-^1^H_ε_ region of ^1^H-^13^C HSQC spectra of ^15^N ^13^C-labeled M2-1_58–177_ in the presence of 0.25 (red) and 0.5 (blue) equivalents of full-length P (22.3 T, 298 K) overlaid on the reference spectrum (black). Broadening is observed for K92 and K158.
(EPS)

Figure S6 **Changes in ^1^H-^15^N HSQC spectra of M2-1_58–177_ single mutants K92D, R126D, T130D, L148A, K150D, R151D and T160D and N163D.** K92D, K150D and R151D strongly reduced *in vitro* RNA binding to M2-1 whereas R126D, T130D, L148A, T160D and N163D were excluded from cytoplasmic inclusion bodies containing RSV phosphoprotein. All resulted in a nearly total loss of transcription in the Luc minigenome assay. (A) ^1^H-^15^N HSQC spectra of ^15^N-labeled M2-1_58–177_ single mutants (125 µM, 18.8 T, 293 K) have a similar pattern to that of wild type and show that the mutant proteins are well folded. (B) Chemical shift difference plots relative to WT M2-1_58–177_ indicate that chemical shift changes occur only for residues close to the mutation in the sequence, as expected if no conformational rearrangement takes place. In the case of K92D (in helix α2), the spatially close region around K150 (in helix α5) is also affected, and vice versa. A ^15^N NOESY-HSQC experiment with 80 ms mixing time was recorded with K150D to verify the presence of H_N,i_-H_N,i+1_ correlations, characteristic of α-helices.
(EPS)

Figure S7 **Sequence alignment of M2-1 and VP30 proteins.** (A) Alignment of primary sequences of M2-1 proteins of human Respiratory Syncytial Virus (hRSV, strain Long, UniProt accession number P28887.1), bovine RSV (bRSV, strain ATue51908, NC_001989.1), Pneumonia virus of mouse (PVM, YP_173333), human Metapneumovirus (HMPV, AAM12941.1), and avian Metapneumovirus (AMPV, YP_443841). Strictly identical residues are highlighted in red. Residues with more than 70% similarity are boxed in blue. Secondary structures extracted from the M2-1_58–177_ structure are indicated above each bloc. Single mutated M2-1_58–177_ residues which severely impair RSV transcription are indicated by a red star if the residues are strictly conserved and a blue star if they diverge in more than 2 sequences. (B) The primary sequences of M2-1 proteins were manually aligned with VP30 protein sequences of Zaire Ebola virus (EBOV_Z,UNP Q05323), Reston EBOV (EBOV_R, NP_690585.1) and Lake Victoria Marburg virus (MARV_LV, YP_001531157.1). Strictly identical residues are highlighted in red and located in the Cys_3_His putative zinc finger and in the oligomerisation domains. Residues with more than 70% similarity are boxed in blue. (C) Primary sequences of VP30 proteins were aligned separately from M2-1 and secondary structures extracted for EBOV_Z VP30 CTD (pdb 2I8B). Alignments were edited with ESPript 2.2 [60].
(EPS)

Text S1 **Supplemental Materials and Methods.**
(DOC)

Table S1 **NMR structure statistics of RSV M2-1_58–177_.**
(DOC)
