# Supplementary figures and images for: Structure and Functional Analysis of the RNA- and Viral Phosphoprotein-Binding Domain of Respiratory Syncytial Virus M2-1 Protein
Source: PLoS Pathog. 2012 May 31;8(5):e1002734. doi: 10.1371/journal.ppat.1002734 (PMC3364950; doi:10.1371/journal.ppat.1002734)

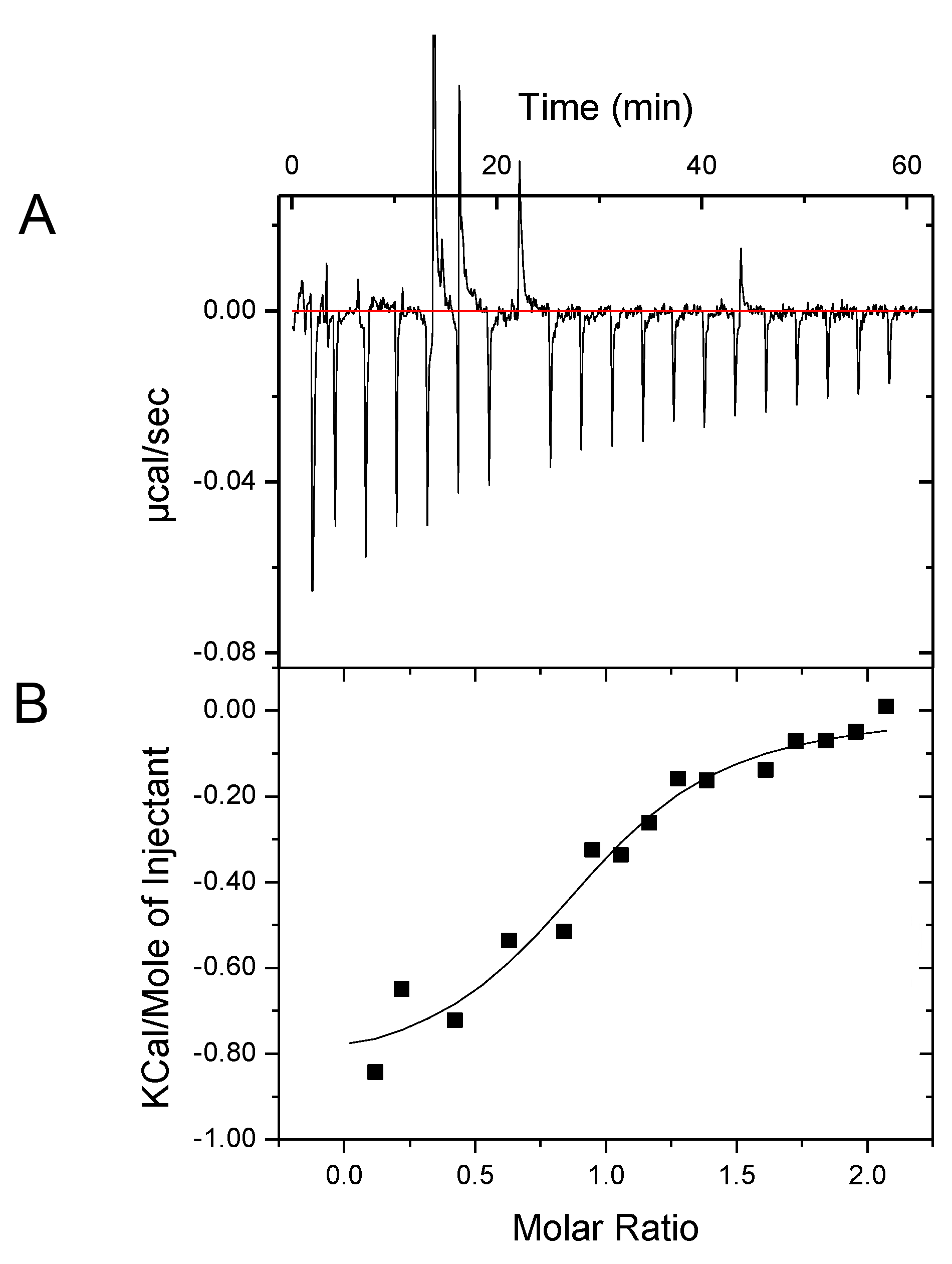

Supplement: Figure S4 — ITC binding isotherms for P binding to M2-158–177. (A) Raw binding data obtained for 20 automatic injections of M2-158–177 (2 µl for each injection, 335 µM protein concentration) into a cell containing P (200 µL initial volume; 33 µM initial protein concentration). Proteins were suspended in 1× PBS. (B) Integrated titration curve obtained from the raw data in panel A after baseline subtraction. The solid squares represent the experimental data, while the solid line corresponds to the standard multiple independent binding-site model that was fitted to the data. The corresponding average Kd value is 3 µM with a stoichiometry of 1∶1. (TIF) [file ppat.1002734.s004.tif]
