# Supplementary material for: Structure and Functional Analysis of the RNA- and Viral Phosphoprotein-Binding Domain of Respiratory Syncytial Virus M2-1 Protein
Source: PLoS Pathog. 2012 May 31;8(5):e1002734. doi: 10.1371/journal.ppat.1002734 (PMC3364950; doi:10.1371/journal.ppat.1002734)
Supplement: Text S1 — Supplemental Materials and Methods. (DOC) [file ppat.1002734.s008.doc]

**Supplemental Materials and Methods**

**Expression and purification of recombinant proteins M2-1 and P**

The cDNA encoding residues 58-177 of M2-1 (M2-1_58-177_) was amplified by PCR with high fidelity PfuTurbo Polymerase (5U, Stratagene), 10 ng of the pGEX-M2-1 plasmid as a matrix as described in , and 100 ng of the following primers: M2-1-58+: 5’-GAGGGATCCGAAATAAGTGGAGCTGCAGAG-3’; M2-1-177-: 5’-TTATTTTGGGTTGTTGATGGTTATGCTC-3’. PCR-amplified DNA was digested by *Bam*HI and inserted at the *Bam*HI-*Sma*I sites in pGEX-4T3 vector (Pharmacia) to engineer the pGEX-M2-1_58-177_ plasmid. This was designed to produce a protein that contained GST fused to residues 58-177 of M2-1 separated by a thrombin cleavage site. Thrombin cleavage of the fusion protein resulted in the addition of two amino acids, a glycine and a serine at the amino terminus of M2-1_58-177_.

M2-1 amino acid substitution mutants were obtained with the Quickchange site-directed mutagenesis kit (Stratagene) by using pGEX-M2-1, pGEX-M2-1_58-177_ and pM2-1 as templates (primer sequences available upon demand). All constructs were sequenced in their entirety to confirm their structure.

*E. coli* BL21 (DE3) (Novagen) cells transformed with the pGEX-M2-1-derived plasmids, pGEX-P or pGEX-P_100-166_ were grown at 37°C for 8 hours in LB medium containing 100 µg/ml ampicillin. The same volume of LB was then added and protein expression was induced by adding 80µg/ml isopropyl-ß-D-thio-galactoside (IPTG) to the medium. The bacteria were incubated at 28°C and harvested by centrifugation 15 h after induction.

Uniformly ^15^N and/or ^13^C-labeled M2-1_58-177_ was prepared by growing transformed bacteria in minimal M9 medium supplemented with 1 g/L ^15^NH_4_Cl (Cortecnet, France) and 4 g/L ^13^C glucose (Cortecnet, France). A 50 mL preculture grown overnight to saturation in LB medium containing 100 μg/mL ampicillin was inoculated into 1 L of minimal M9 medium supplemented with ampicillin. The culture was grown at 37°C for 8 hours. The same volume of M9 medium was then added and protein expression was induced by adding 80 µg/mL isopropyl-ß-D-thio-galactoside (IPTG) to the medium. The bacteria were incubated for a further 15 hours at 28°C and harvested by centrifugation. Bacterial pellets were resuspended in 100 ml of lysis buffer (50 mM Tris-HCl pH 7.4, 1 M NaCl, 1 mM DTT, 2% Triton X-100, 10 mM MgSO_4_, 1 mM CaCl_2_, 1 mg/mL lysozyme) supplemented with a complete protease inhibitor cocktail (Roche, Mannheim, Germany) and incubated for 1 hour on ice, sonicated and centrifuged twice at 4°C for 30 min at 10,000 g. Glutathione-Sepharose 4B beads (GE Healthcare) were added to the clarified supernatants (2 mL of beads for 1 liter of induced bacteria culture) and incubated at 4°C for 15 hours. Beads were washed three times with lysis buffers and then stored at 4°C in an equal volume of lysis buffer. To isolate GST-free proteins, beads containing bound GST-fusion proteins were extensively washed with PBS 1X + 1 mM DTT, resuspended in an equal volume of PBS + 1 mM DTT and incubated with biotinylated thrombin (Novagen) for 16 hours at 20°C. Thrombin was then removed using the Thrombin cleavage capture kit according to the manufacturer’s instructions (Novagen).

^2^H^15^N labeled M2-1_58-177_ was produced with a similar protocol in ^2^H-M9 medium, prepared with 99% D_2_O (Eurisotop, France) and supplemented with 1 g/L ^15^NH_4_Cl and 2 g/L ^2^H-labeled glucose. A 100 mL preculture in LB was used to inoculate 1 L of unlabeled M9. After 2 h of incubation, the unlabeled culture was centrifuged and then resuspended in 100 mL ^2^H-M9. After 1 h of growth, the culture was again centrifuged, the pellet resuspended in 900 mL ^2^H-M9 and expression induced for 15 h at 28°C.

**Structure calculation**

Structures of M2-1_58-177_ were calculated with CYANA 2.1 with automated NOE peak assignment and dihedral angle constraints calculated from the backbone chemical shifts with the program TALOS. 20 conformers generated during the seventh cycle of CYANA were further refined by simulated annealing using the IVM module in Xplor-NIH . The experimental terms of the target function consisted of NOE-derived upper-limit distance constraints generated by CYANA, dihedral angle constraints and ^1^D_NH_ residual dipolar coupling constraints with final force constants of 30 kcal mol^-1^ Å^-2^, 200 kcal mol^-1^ rad^-2^ and 0.5 kcal mol^-1^ Hz^-2^ respectively. Alignment tensor parameters were allowed to float all over the protocol. Force constants for bonds and angular terms were set to 1000 kcal mol^-1^ Å^-2^ and 500 kcal mol^-1^ rad^-2^ respectively. Nonbonded interactions were described by a quartic van der Waals repulsion term (final force constant = 4 kcal mol^-1^ Å^-4^, van der Waals radius scale factor = 0.8). High temperature dynamics were performed at 3000 K for 10 ps with the RDC force constant set to 0.01 kcal mol^-1^ Hz^-2^. The bath was cooled during the dynamics run to 25 K with 12.5 K steps and a 0.2 ps internal coordinates dynamics run performed at each temperature. Final minimization was conducted with a 500-step torsion angle minimization followed by a 100-step Cartesian minimization. The final ensemble of 20 structures was selected from a total of 100. Structure statistics are summarized in Table 1. Overall quality was assessed by PROCHECK-NMR and on the Protein Structure Validation Suite server (http://psvs-1_4-dev.nesg.org/). Visualization and graphic rendering of the protein structures were performed with PyMOL .

**SI references**

1. Tran TL, Castagne N, Dubosclard V, Noinville S, Koch E, et al. (2009) The respiratory syncytial virus M2-1 protein forms tetramers and interacts with RNA and P in a competitive manner. J Virol 83: 6363-6374.

2. Castagne N, Barbier A, Bernard J, Rezaei H, Huet JC, et al. (2004) Biochemical characterization of the respiratory syncytial virus P-P and P-N protein complexes and localization of the P protein oligomerization domain. J Gen Virol 85: 1643-1653.

3. Herrmann T, Guntert P, Wuthrich K (2002) Protein NMR structure determination with automated NOE assignment using the new software CANDID and the torsion angle dynamics algorithm DYANA. J Mol Biol 319: 209-227.

4. Schwieters CD, Kuszewski JJ, Clore GM (2006) Using Xplor–NIH for NMR molecular structure determination. Prog NMR Spectrosc 48: 47-62.

5. Laskowski RA, Rullmannn JA, MacArthur MW, Kaptein R, Thornton JM (1996) AQUA and PROCHECK-NMR: programs for checking the quality of protein structures solved by NMR. J Biomol NMR 8: 477-486.

6. Schrodinger L (2010) The PyMOL Molecular Graphics System, version 1.3. Available: http://www.pymol.org.
