# Supplementary material for: Structure and Functional Analysis of the RNA- and Viral Phosphoprotein-Binding Domain of Respiratory Syncytial Virus M2-1 Protein
Source: PLoS Pathog. 2012 May 31;8(5):e1002734. doi: 10.1371/journal.ppat.1002734 (PMC3364950; doi:10.1371/journal.ppat.1002734)
Supplement: Table S1 — NMR structure statistics of RSV M2-158–177. (DOC) [file ppat.1002734.s009.doc]

**Table S1: NMR structure statistics of RSV M2-1_58-177_**

| **Number of NOE distance constraints** | | 1539 |  |
| --- | --- | --- | --- |
|  | Intraresidual | 515 |  |
|  | Sequential | 405 |  |
|  | Medium range: 1 <|i - j| <5 | 365 |  |
|  | Long range: |i - j| ≥ 5 | 254 |  |
| **Number of dihedral angle constraints**^a^ | | 151 | 77Φ, 74Ψ |
| **Number of residual dipolar couplings**^b^ | | 100 |  |
|  | ^1^D_NH_(C12E5:hexanol) | 56 |  |
|  | ^1^D_NH_(stretched gel) | 44 |  |
| **Violation Statistics** (mean ± sd)^c^ | |  |  |
|  | Residual distance violations > 0.3 Å/structure (Å) | 0.5 ± 0.6 |  |
|  | Residual dihedral angle violations >5° (°) | 0.0 ± 0.0 |  |
|  | Maximum distance violation (Å) | 0.35± 0.02 |  |
|  | Maximum dihedral angle violation (Å) | 3.5 ± 0.5 |  |
|  | RMSD from NOE contraints (Å) | 0.02 ± 0.01 |  |
|  | RMSD from dihedral angle constraints (Hz) | 0.47 ± 0.03 |  |
|  | RMSD from rdc (c12e5) (Hz) | 0.47 ± 0.02 |  |
|  | RMSD from rdc (gel) (Hz) | 1.07 ± 0.03 |  |
| **Rmsd from ideal covalent geometry** (mean ± sd)^c^ | |  |  |
|  | Bonds (Å) | 0.002 ± 0.000 |  |
|  | Angles (°) | 0.427 ± 0.005 |  |
|  | Improper torsions (°) | 0.270 ± 0.006 |  |
| **Alignment tensor**^d^ (mean ± sd)^c^ | | **D_a_ (Hz)** | **R** |
|  | C12E5:hexanol | 20.7 ± 0.6 | 0.17 ± 0.03 |
|  | Stretched gel | 8.8 ± 0.2 | 0.42 ± 0.02 |
| **Ramachandran plot statistics**^e^ | | **residues 59-177** | **residues 76-171** |
|  | Most favored | 81.9% | 92.7% |
|  | Additionally allowed | 12.7% | 5.3% |
|  | Generously allowed | 4.7% | 2.0% |
|  | Disallowed | 0.6% | 0% |
| **Rmsd to mean coordinates** | |  |  |
|  | Backbone (76-171)^f^ (Å) | 0.54 ± 0.06 |  |
|  | Heavy atoms (76-171)^f^ (Å) | 0.99 ± 0.07 |  |

^a^Determined from backbone chemical shifts by TALOS .

^b15^N-^1^H RDCs measured in two alignment media (C12E5:hexanol and stretched polyacrylamide gel) were added as restraints for structure refinement with XPLOR-NIH .

^c^Mean and standard deviation values were calculated from the 20 lowest energy structures ensemble.

^d^D_a_: magnitude of residual dipolar coupling tensor, R: rhombicity.

^e^Calculated with Procheck-nmr for the 20 structure ensemble, for all residues (59-177) and the ordered core domain (76-171).

^f^Calculated for the ordered core domain (76-171).

**References:**

1. Cornilescu G, Delaglio F, Bax A (1999) Protein backbone angle restraints from searching a database for chemical shift and sequence homology. J Biomol NMR 13: 289-302.

2. Schwieters CD, Kuszewski JJ, Tjandra N, Clore GM (2003) The Xplor-NIH NMR molecular structure determination package. J Magn Reson 160: 65-73.

3. Laskowski RA, Rullmannn JA, MacArthur MW, Kaptein R, Thornton JM (1996) AQUA and PROCHECK-NMR: programs for checking the quality of protein structures solved by NMR. J Biomol NMR 8: 477-486.
